# Expected accuracy of proximal and distal temperature estimated by wireless sensors, in relation to their number and position on the skin

**DOI:** 10.1371/journal.pone.0180315

**Published:** 2017-06-30

**Authors:** Enrico Longato, Maria Garrido, Desy Saccardo, Camila Montesinos Guevara, Ali R. Mani, Massimo Bolognesi, Piero Amodio, Andrea Facchinetti, Giovanni Sparacino, Sara Montagnese

**Affiliations:** 1 Department of Information Engineering, University of Padua, Padua, Italy; 2 Department of Medicine, University of Padua, Padua, Italy; 3 Division of Medicine, University College London, London, United Kingdom; University of Fribourg, SWITZERLAND

## Abstract

A popular method to estimate proximal/distal temperature (*T*_*PROX*_ and *T*_*DIST*_) consists in calculating a weighted average of nine wireless sensors placed on pre-defined skin locations. Specifically, *T*_*PROX*_ is derived from five sensors placed on the infra-clavicular and mid-thigh area (left and right) and abdomen, and *T*_*DIST*_ from four sensors located on the hands and feet. In clinical practice, the loss/removal of one or more sensors is a common occurrence, but limited information is available on how this affects the accuracy of temperature estimates. The aim of this study was to determine the accuracy of temperature estimates in relation to number/position of sensors removed. Thirteen healthy subjects wore all nine sensors for 24 hours and reference *T*_*PROX*_ and *T*_*DIST*_ time-courses were calculated using all sensors. Then, all possible combinations of reduced subsets of sensors were simulated and suitable weights for each sensor calculated. The accuracy of *T*_*PROX*_ and *T*_*DIST*_ estimates resulting from the reduced subsets of sensors, compared to reference values, was assessed by the mean squared error, the mean absolute error (MAE), the cross-validation error and the 25^th^ and 75^th^ percentiles of the reconstruction error. Tables of the accuracy and sensor weights for all possible combinations of sensors are provided. For instance, in relation to *T*_*PROX*_, a subset of three sensors placed in any combination of three non-homologous areas (abdominal, right or left infra-clavicular, right or left mid-thigh) produced an error of 0.13°C MAE, while the loss/removal of the abdominal sensor resulted in an error of 0.25°C MAE, with the greater impact on the quality of the reconstruction. This information may help researchers/clinicians: *i)* evaluate the expected goodness of their *T*_*PROX*_ and *T*_*DIST*_ estimates based on the number of available sensors; *ii)* select the most appropriate subset of sensors, depending on goals and operational constraints.

## Introduction

Skin temperature has been comprehensively studied from a chronobiological stand point, with particular attention to its rhythm in relation to the onset of sleep [1–5].

Contactless infrared and conductive devices are the most common tools used for measuring skin temperature [6–8]. Contactless infrared thermometers and infrared thermography have proven effective in diverse settings [6,8,9]. However, they are difficult to use in free-living conditions, for long periods of continuous acquisition [6,8,9]. Conductive devices are cheaper and generally easier to use [9–15]. They can be divided into two categories, based on the presence or absence of wiring. Hard-wired thermistors and thermocouples that are worn on the body may limit subjects’ comfort and mobility [6–8]. In contrast, conductive wireless sensors are unobtrusive to wear [7,16], also in free-living conditions and for long periods of time [7,13,17]. Their limitations are the finite lifetime of their battery and the fact that recordings can only be viewed once complete, thus not allowing for adjustments during acquisition.

As for the number of sites to be considered for skin temperature measurement, several proposals have been put forward, using from 3 to 15 different skin locations [18–27]. From a methodological point of view, one of the most complete studies published so far on the validation of wireless sensors for human use in a clinical/circadian context was carried out by Van Marken Lichtenbelt et al. [7]. These authors used 9 wireless sensors, each with its own weight in formulas which are utilized to obtain distal (*T*_*DIST*_, 4 sensors) and proximal (*T*_*PROX*_, 5 sensors) temperature, based on a modification of the original formulas proposed by Kräuchi et al. [28].

In clinical practice, the loss or removal of one or more sensors is a common occurrence. However, virtually no information is available on the actual impact of the loss/removal of one or more sensors on the accuracy of the temperature estimates. Therefore, the aim of this study was to mathematically determine the expected reliability of temperature estimates in relation to number and position of sensors utilized.

## Methods

### Subjects

A total of 13 healthy volunteers [five males; mean age: 47.3 ± 14.5 (22–65) years] were enrolled. They were excluded if they were under 18 years of age; could not/were unwilling to comply with the study procedures, had misused alcohol in the preceding 6 months, had undertaken shift work or intercontinental travel in the preceding four months, or were on chronic medical treatment.

The study was approved by the Padova University Hospital Ethics Committee (Ref. AOP0536, CESC 3639/AO/15). All participants provided written, informed consent. The study was conducted according to the Declaration of Helsinki (Hong Kong Amendment) and Good Clinical Practice (European) guidelines.

### Data acquisition

Temperature recordings were carried out over 24 hours (from 12:00 midday to 12:00 midday of the following day) by use of temperature sensors (iButton^®^, model no. DS1922L-F5, Maxim Integrated, San Jose, CA, USA). These are made of a semiconductor temperature sensor, a computer chip with a real time clock and memory, and a 3V lithium battery, all enclosed in a 16x6 mm^2^ stainless steel can. Manufacturing specifications include: temperature accuracy of ± 0.5°C from -10 to +65°C, and operating temperature range -40°C to +85°C. Sampling rate was set at 3 min with a resolution of 0.0625°C.

**Fig 1:**
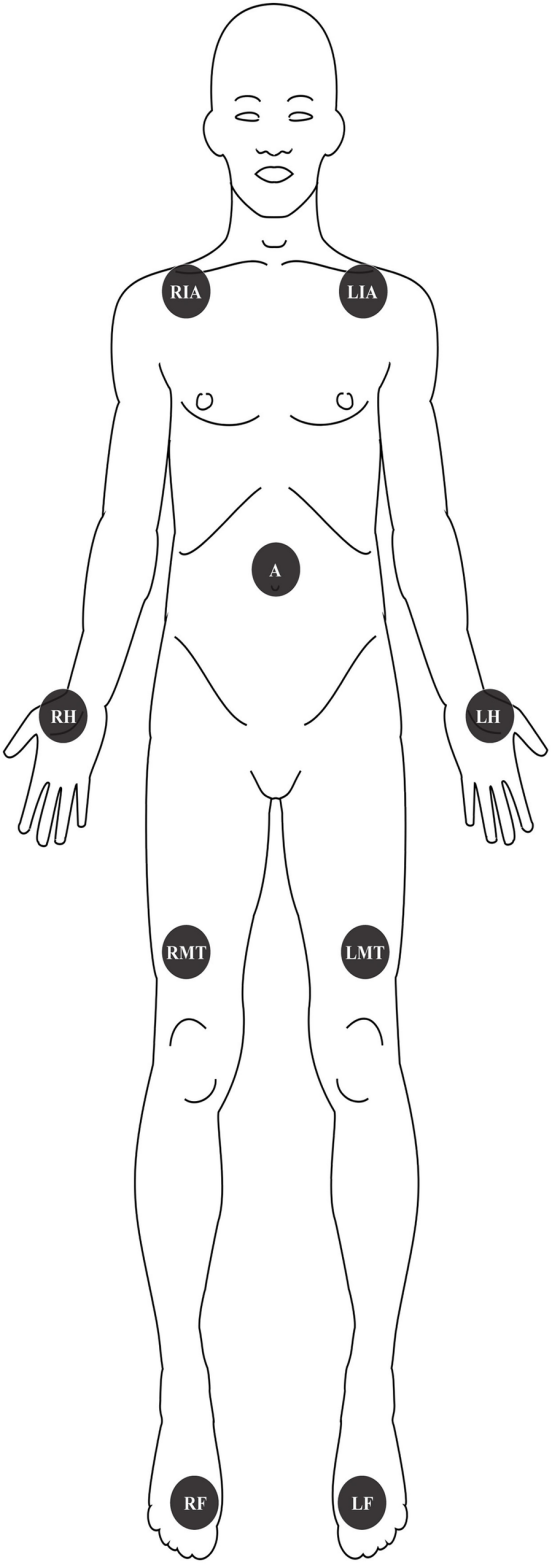
Skin locations of the wireless sensors. LIA: left infra-clavicular area; RIA: right infra-clavicular area; A: abdomen; LH: left hand; RH: right hand; LMT: left mid-thigh; RMT: right mid-thigh; LF: left foot; RF: right foot.

Nine sensors were placed on the skin and secured using medical tape, on the following locations: on the muscular rectus femoris on the left and right mid-thigh (LMT, RMT); left and right infra-clavicular area (LIA, RIA); abdomen (A); thenar area at the palmar sites on the left and right hand (LH, RH), and on the mid metatarsal area at the plantar site of the left and right foot (LF, RF) (Fig 1). Participants were asked to keep the sensors on at all times except when showering/bathing. Data from the sensors were transferred by an adapter (DS1402D) to a computer, using the iButton Viewer software (Dallas Semiconductor, Maxim Integrated Products, Sunnyvale, CA).

### Data analysis

Reference *T*_*PROX*_ and *T*_*DIST*_ temperatures were calculated using all nine sensors as described in the following subsection. Then, all possible combinations of reduced sets of sensors were simulated and, for each of them, the set of weights for *T*_*PROX*_ and *T*_*DIST*_ formulas were recalculated by Linear Least Squares (LLS). Finally, the accuracy of the estimates obtained by using reduced sets of sensors with the pertinent, recalculated weights was quantified by numerical indicators.

### Determination of reference T_DIST_ and T_PROX_

The reference *T*_*PROX*_ was calculated as follows [7]:

$$T_{PROX}=0.324\;T_{A}+0.1915\;T_{LMT}+0.1915\;T_{RMT}+0.1465\;T_{LIA}+0.1465\;T_{RIA}$$ (1)

where *T*_*A*_, *T*_*LIA*_, *T*_*RIA*_, *T*_*LMT*_ and *T*_*RMT*_ are the temperature values acquired by sensors placed on the abdomen, left mid-thigh, right mid-thigh, left infra-clavicular area and right infra-clavicular area, respectively.

Similarly, the reference *T*_*DIST*_ was calculated as follows [7]:

$$T_{DIST}=0.25\;T_{LF}+0.25\;T_{RF}+0.25\;T_{LH}+0.25\;T_{RH}$$ (2)

where *T*_*LF*_, *T*_*RF*_, *T*_*LH*_, and *T*_*RH*_ are the temperature values acquired by sensors placed on the left foot, right foot, left hand, and right hand, respectively.

As it is apparent from Eqs (1) and (2), *T*_*PROX*_ and *T*_*DIST*_ are calculated by multiplying the appropriate sensor data by a fixed set of weights. Should sensors lose synchronization or stop working, the accuracy of *T*_*PROX*_ and *T*_*DIST*_ could become poor. While errors introduced by loss of synchronization or occasional artifacts can be mitigated by pre-processing (see S1 File for the procedure employed by Van Marken Lichtenbelt et al. [7]), the effect of the reduction of the number of sensors on accuracy requires *ad hoc* modeling.

### Estimation of *T*_*PROX*_ and *T*_*DIST*_ from reduced sets of sensors

In order to assess the impact of the loss of one or more sensors, the formulas for calculation of *T*_*PROX*_ and *T*_*DIST*_ were re-defined as follows:

$${\hat{T}}_{PROX}=w_{A}\;T_{A}+w_{LIA}\;T_{LIA}+w_{RIA}\;T_{RIA}+w_{LMT}\;T_{LMT}+w_{RMT}\;T_{RMT}$$ (3)

$${\hat{T}}_{DIST}=w_{LF}\;T_{LF}+w_{RF}\;T_{RF}+w_{LH}\;T_{LH}+w_{RH}\;T_{RH}$$ (4)

Where ${\hat{T}}_{\mathrm{PROX}}$ and ${\hat{T}}_{\mathrm{DIST}}$ are the best estimates of *T*_*PROX*_ and *T*_*DIST*_ which can be obtained from a reduced number of sensors provided that, for each combination of the available sensors, the ‘optimal’ set of weights is numerically determined by LLS, i.e. by minimizing the sum of square differences between the values of the estimates, obtained by either (3) or (4), and the corresponding target references, obtained by either (1) or (2). In practice, all the data measured by sensors placed in corresponding locations on each patient were combined into a single macro-signal (signals belonging to each patient are appended to the respective macro-signal in the same order) of size *N* (here, after the pre-processing described in S1 File, N = 5256). Then, matrix ***X*** (size *N×P*) was constructed by storing, in *P* separate columns, the *P* macro-signals corresponding to the considered subset of *P* sensors. Letting vector ***T*** denote the *N*-size vector of the reference values, the *P*-sized vector ***ŵ*** of the ‘optimal’ weights for the considered subset of *P* sensors, obtained by LLS, obeys the formula:

$$\hat{w}={\left(X\prime \;X\right)}^{-1}X\prime \;T$$ (5)

Using these weights, the best reconstruction, in the LLS sense, of the *N* temperature values contained in vector ***T*** that is possible to achieve using the subset of signals stored in ***X*** is:

$$\hat{T}=X\;\hat{w}$$ (6)

For example, should the RMT sensor be unavailable for determination of *T*_*PROX*_, in Eq (3) *w*_*RMT*_ *= 0* and the adjusted optimal values of *w*_*A*_, *w*_*LI*_, *w*_*RIA*_ and *w*_*LMT*_ are obtained by minimizing the sum of the square differences between the predictions of the model ${\hat{T}}_{\mathrm{PROX}}$ given by Eq (3) and the reference *T*_*PROX*_ given by Eq (1). In this case, matrix ***X*** has *N* rows and 4 columns (containing all the recordings A, LIA, RIA and LMT of all the subjects) and ***ŵ*** *= [ŵ*_*A*_, *ŵ*_*LIA*_, *ŵ*_*RIA*_, *ŵ*_*LMT*_*]*^*T*^ is the column vector of the (re-tuned) values of the weights calculated by Eq (5). Having calculated **ŵ**, the estimate $\hat{T}$ that most closely resembles **T**, despite not including data from the RMT sensor, can be obtained using formula (6) which means that, at any given point in time, the value of ${\hat{T}}_{\mathrm{PROX}}$ can be obtained as:

$${\hat{T}}_{PROX}={\hat{w}}_{A}\;T_{A}+{\hat{w}}_{LIA}\;T_{LIA}+{\hat{w}}_{RIA}\;T_{RIA}+{\hat{w}}_{LMT}\;T_{LMT}$$ (7)

### Accuracy of the estimates obtained from reduced sets of sensors

To quantitatively assess the goodness of the estimates obtained by Eq (6), the following indicators were considered:

1. the mean squared error (MSE, which, apart from a scale factor, coincides with the minimal value of the cost function employed in the LLS procedure):
  $$MSE=\frac{1}{N}{\left|\left|T-\hat{T}\right|\right|}^{2}$$ (8)
2. the mean absolute error (MAE) between the reference and the estimate:
  $$MAE=\frac{1}{N}\sum_{i=1}^{N}\left|T_{i}-{\hat{T}}_{i}\right|$$ (9)
  Where *T*_*i*_ and ${\hat{T}}_{i}$ denotethe i-th element of the N-dimensional vector ***T*** and $\hat{T}$, respectively.
3. the (absolute) cross-validation error (CVE) obtained by estimating the reconstructed $\hat{T}$ relative to each subject using only the data from the other 12 subjects. In practice, this entails estimating 13 sets of weights on different portions of the data and using each set to reconstruct whichever portion was not used to calculate the said set.
4. the quartiles Q1 and Q3, corresponding to the 25^th^ and 75^th^ percentile, of the (signed) estimation error.

## Results

### Proximal temperature

The whole set of estimated weights ***ŵ*** for each possible combination of sensors, together with the pertinent performance factors are presented in Table 1. It should be noted that the number *P* of unknown parameters, i.e. the size of ***ŵ***, is low compared to the number *N* of data, suggesting that results are not expected to be affected by the sample size. The values of MSE, MAE, Q1 and Q3 reported in Table 1 for each combination of sensors can be used to assess which degree of deterioration is to be expected for *T*_*PROX*_ estimation in case one/more sensors are missing.

**Table 1:** Performance factors and weights to be assigned to each sensor to estimate *T*_*PROX*_, relative to each possible combination of sensors.

	Performance Factors					Sensor Weights				
	MSE	MAE	CVE	Q1	Q3	A	LIA	RIA	LMT	RMT
N (Available Sensors)	(Mean Squared Error)	(Mean Absolute Error)	(Cross-Validation Error)	(25th percentile)	(75th percentile)	(Abdomen)	(Left Infra-clavicular Area)	(Right Infra-clavicular Area)	(Left Mid-Thigh)	(Right Mid-Thigh)
1	0.688	0.677	0.714	-0.557	0.610	0.981	0	0	0	0
1	1.332	0.936	0.968	-0.745	0.862	0	0.971	0	0	0
1	1.229	0.893	0.916	-0.686	0.809	0	0	0.971	0	0
1	1.909	1.091	1.127	-0.904	0.894	0	0	0	1.036	0
1	2.005	1.106	1.144	-0.941	0.762	0	0	0	0	1.039
2	0.590	0.621	0.662	-0.436	0.558	0.720	0.259	0	0	0
2	0.563	0.604	0.641	-0.434	0.553	0.685	0	0.293	0	0
2	0.148	0.300	0.307	-0.226	0.257	0.632	0	0	0.370	0
2	0.131	0.285	0.296	-0.218	0.250	0.635	0	0	0	0.367
2	1.165	0.864	0.899	-0.647	0.789	0	0.372	0.599	0	0
2	0.162	0.319	0.330	-0.265	0.268	0	0.535	0	0.467	0
2	0.184	0.330	0.353	-0.256	0.264	0	0.542	0	0	0.461
2	0.169	0.324	0.343	-0.269	0.275	0	0	0.546	0.455	0
2	0.195	0.341	0.367	-0.252	0.283	0	0	0.554	0	0.448
2	1.767	1.046	1.099	-0.882	0.772	0	0	0	0.585	0.453
3	0.562	0.604	0.655	-0.430	0.555	0.678	0.045	0.255	0	0
3	0.035	0.136	0.146	-0.099	0.101	0.347	0.277	0	0.375	0
3	0.032	0.138	0.144	-0.121	0.102	0.372	0.260	0	0	0.367
3	0.033	0.142	0.151	-0.101	0.122	0.351	0	0.281	0.366	0
3	0.032	0.133	0.136	-0.097	0.107	0.379	0	0.262	0	0.359
3	0.114	0.263	0.279	-0.204	0.230	0.619	0	0	0.162	0.223
3	0.131	0.287	0.301	-0.233	0.233	0	0.285	0.266	0.450	0
3	0.154	0.304	0.325	-0.232	0.245	0	0.297	0.261	0	0.443
3	0.134	0.286	0.313	-0.222	0.234	0	0.524	0	0.274	0.206
3	0.144	0.298	0.328	-0.242	0.254	0	0	0.535	0.276	0.192
4	0.025	0.121	0.133	-0.096	0.105	0.325	0.144	0.160	0.370	0
4	0.024	0.115	0.119	-0.094	0.087	0.353	0.141	0.143	0	0.363
4	0.009	0.067	0.074	-0.054	0.049	0.344	0.269	0	0.190	0.198
4	0.009	0.070	0.076	-0.042	0.058	0.351	0	0.270	0.189	0.190
4	0.106	0.254	0.280	-0.203	0.212	0	0.287	0.252	0.268	0.194
5	0	0	0	0	0	0.324	0.1465	0.1465	0.1915	0.1915

As it can be observed by comparing each value of MSE to its corresponding MAE, both indicators follow the same trend, even though MSE amplifies differences in goodness of reconstruction between combinations of the same number of sensors. As expected, using all sensors (*P =* 5) brings to MSE = 0, and in this case the estimated weights are the same as those of the original *T*_*PROX*_ formula. Conversely, using data from one sensor (*P =* 1) results in higher MSE values, and in the least accurate possible reconstruction. As shown by consistently higher values of MSE in Table 1, the loss/removal of the abdominal (A) sensor tends to have the greatest impact on the reconstruction (Table 1, column 2, *w*_*A*_ = 0).

As expected, Table 1 also shows that both MAE and the difference between Q3 and Q1 diminish as the number of working sensors increases. Using only one sensor (*P =* 1), wherever it may be placed, results in a less accurate assessment of *T*_*PROX*_ (MAE up to 1.1°C), while the use of the entire set of sensors (*P* = 5) allows to get a perfect reconstruction, consistently with what was already discussed in relation to MSE.

The robustness of the presented results can be appreciated by comparing the MAE and CVE values relative to each sensor combination: as it can be observed, the difference between the two indicators is negligible. The most important implication of this finding is the fact that the goodness of reconstruction of ***T***_*PROX*_ remains consistent even when ***ŵ*** is used on previously unseen temperature readings. In other words, the sample size available for the estimation process is sufficiently large to warrant robustness of the results. This is further confirmed by the negligible differences between MAE and CVE for each sensor combination: if 12 subjects were not sufficient to estimate the whole temperature signal relative to the 13^th^, one we expect much larger CVE values.

**Fig 2:**
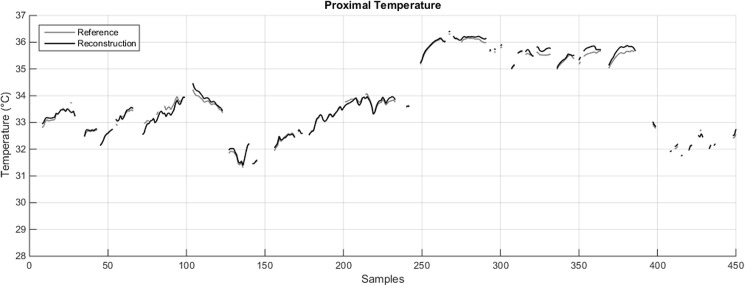
Comparison between the reference *T*_*PROX*_ (grey line) and its reconstruction ${\hat{T}}_{\mathrm{PROX}}$ (black line) obtained in one subject with three sensors (A, RIA, RMT) worn for 22.5 hours, weighed by the appropriate coefficients provided in Table 1. The gaps in the plot are the result of the pre-processing procedure described in S1 File.

Should the number of available sensors for estimating *T*_*PROX*_ be pre-defined (and lower than 5), results reported in Table 1 also allow to speculate on the preferred locations where such sensors should be placed, and the way they should be weighted to reconstruct *T*_*PROX*_. For instance, if only *P = 3* sensors were available, the best fidelity to the references is expected to be achieved by placing the sensors in any combination of three non-homologous areas (A, RIA or LIA, RMT or LMT). This is expected to allow the estimation of *T*_*PROX*_ with as little as 0.13°C of MAE, which is in line with the error intrinsic to each sensor (Table 1, column 3, 3 available sensors, grey cells). As the iButton resolution is 0.0625°C and resolution is necessarily the lower bound to measurement accuracy, MAE values of about double the resolution are comparable with sensor accuracy. An example of reconstruction based on three sensors placed on three non-homologous areas is presented in Fig 2, where it is apparent how closely the reference and newly-estimated signals match.

### Distal temperature

The general considerations made in relation to *T*_*PROX*_, including those on sample size (*N* = 5477 in the case of *T*_*DIST*_), hold true for *T*_*DIST*_. The main difference lies in the fact that there is no *T*_*DIST*_ equivalent to the abdominal sensor, i.e. the one with no homologue. Table 2 shows how it seems important that at least one of each pair of homologue sensors works correctly, with MSE being consistently higher when either both hands or both feet cannot be monitored (Table 2, column 2, 2 available sensors, grey cells).

**Table 2:** Performance factors and weights to be assigned to each sensor to estimate *T*_*DIST*_, relative to each possible combination of sensors.

	Performance Factors					Sensor Weights			
	MSE	MAE	CVE	Q1	Q3	LF	RF	LH	RH
N (Available Sensors)	(Mean Squared Error)	(Mean Absolute Error)	(Cross-Validation Error)	(25th percentile)	(75th percentile)	(Left Foot)	(Right Foot)	(Left Hand)	(Right Hand)
1	1.318	0.830	0.848	-0.607	0.561	1.018	0	0	0
1	0.983	0.706	0.710	-0.434	0.519	0	1.017	0	0
1	1.322	0.805	0.813	-0.504	0.557	0	0	0.980	0
1	1.075	0.762	0.777	-0.522	0.582	0	0	0	0.982
2	0.960	0.691	0.707	-0.437	0.483	0.205	0.812	0	0
2	0.107	0.241	0.248	-0.186	0.166	0.510	0	0.490	0
2	0.094	0.223	0.233	-0.158	0.164	0.481	0	0	0.519
2	0.085	0.215	0.225	-0.155	0.168	0	0.550	0.451	0
2	0.106	0.239	0.246	-0.154	0.193	0	0.522	0	0.479
2	0.925	0.689	0.709	-0.406	0.536	0	0	0.373	0.608
3	0.057	0.172	0.181	-0.116	0.122	0.226	0.323	0.453	0
3	0.066	0.180	0.186	-0.107	0.129	0.270	0.246	0	0.484
3	0.028	0.119	0.128	-0.086	0.089	0.464	0	0.248	0.286
3	0.034	0.131	0.139	-0.097	0.100	0	0.504	0.260	0.236
4	0	0	0	0	0	0.2500	0.2500	0.2500	0.25000

**Fig 3:**
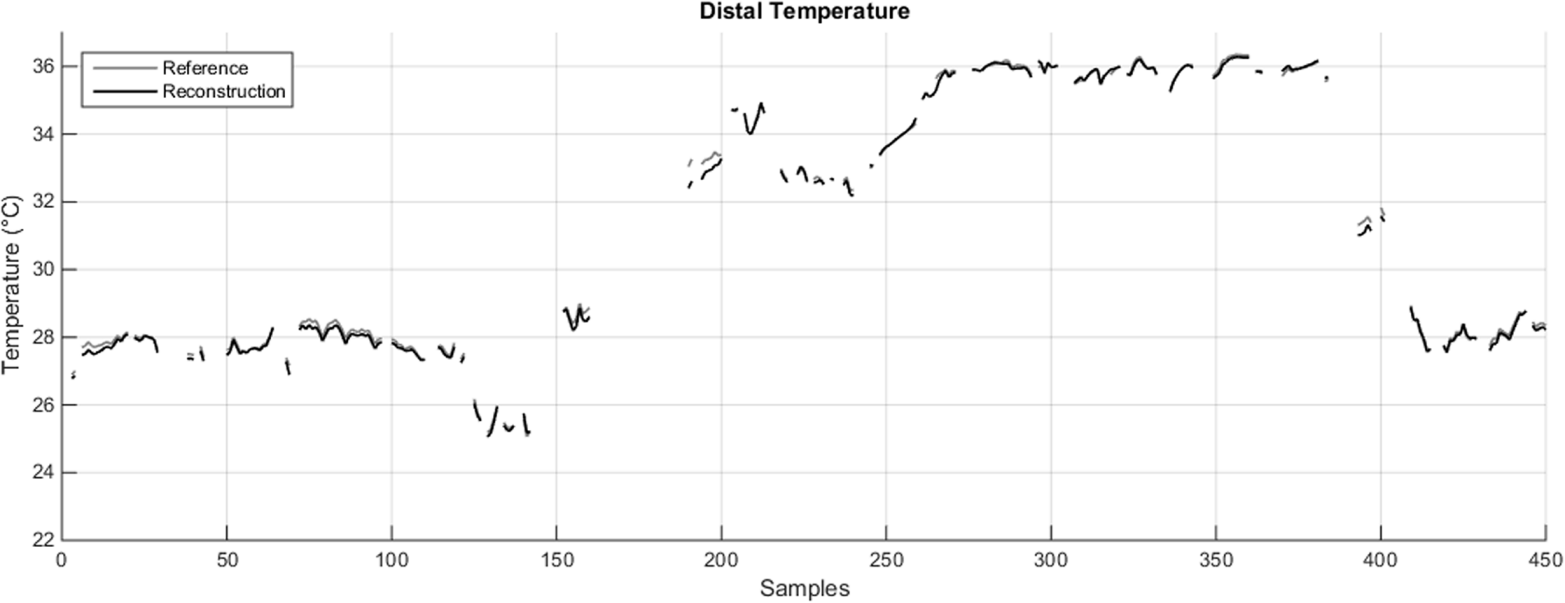
Comparison between the reference *T*_*DIST*_ (grey line) and its reconstruction ${\hat{T}}_{\mathrm{DIST}}$ (black line) obtained in one subject with three sensors (LF, LH, RH) worn for 22.5, weighed by the appropriate coefficients provided in Table 2. The gaps in the plot are the result of the pre-processing procedure described in S1 File.

As it is the case with *T*_*PROX*_, it is possible to speculate that a reasonably accurate estimate of *T*_*DIST*_ can still be obtained by using three sensors, especially when both hands are included (MAE = 0.12–0.13°C; Table 2, column 3, 3 available sensors, grey cells). Fig 3 shows the *T*_*DIST*_ reconstruction achieved with such a configuration for the same subject whose *T*_*PROX*_ was presented in Fig 2. Again, the signals almost perfectly match.

### Final methodological remarks

**Fig 4:**
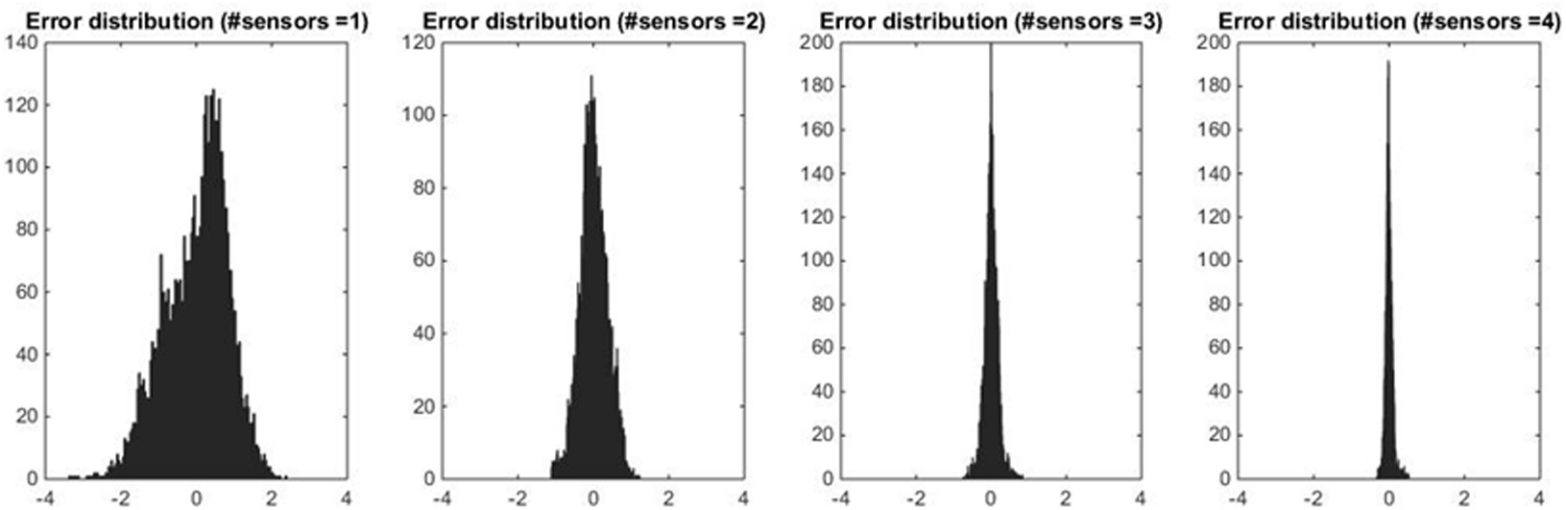
Error distribution in the reconstruction of *T*_*PROX*_. From left to right, the histograms show the absolute frequency of the errors for the ‘best’ configurations of 1, 2, 3 and 4 sensors.

In principle, MAE, Q1 and Q3 could not be adequately representative of multimodal or particularly skewed error distributions. Therefore, as an additional guarantee of method appropriateness, the error distribution for the best combination of 1, 2, 3, 4 (and 5 in the case of *T*_*PROX*_) sensors was also examined. Fig 4 shows how the error distribution is indeed unimodal, zero-mean and symmetrical, confirming that MAE, Q1 and Q3 provide an appropriate representation of its properties. Moreover, it can be inferred that the reconstruction of *T*_*PROX*_ using the newly-estimated weights ***ŵ*** is not prone to overestimation or underestimation, especially when the number of sensors is adequate.

**Fig 5:**
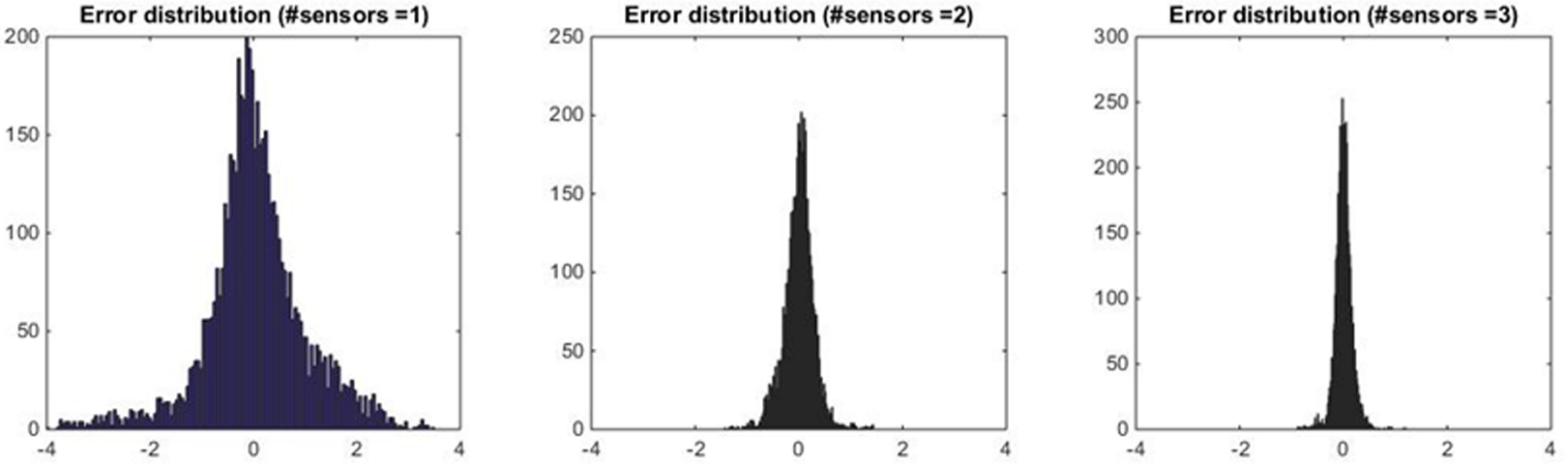
Error distribution in the reconstruction of *T*_*DIST*_. From left to right, the histograms show the absolute frequency of the errors for the ‘best’ configurations of 1, 2, and 3 sensors.

Fig 5 confirms that the same considerations apply to *T*_*DIST*_, which exhibits the same behavior as *T*_*PROX*_. Similarly, the error distribution shows improvement in symmetry as the number of sensors increases.

As a final check and in order to assess whether reconstruction quality could be affected by circadian temperature variations, MAE was calculated separately for samples collected during the “day” (7 am—7 pm) and at “night” (7 pm—7 am) for all combinations of sensors. MAE in the two time intervals was very similar for ‘good’, i.e. low MSE, combinations of sensors. In the case of higher MSE combinations, “night” estimates seemed to be generally less affected by reconstruction errors than their “day” counterparts.

## Discussion

Wireless sensors are a relatively unobtrusive, simple and reliable tool to measure skin temperature. However, the use of a full set of 9 sensors [7] for prolonged periods of time may represent a problem, both in active individuals (for example because the sensors may interfere with certain routine activities) and in elderly people or in patients, who may be unable to re-position a sensor that has been removed. Thus variations in the number of sensors and skin locations have been put forward, with as few as one sensor (on the foot or on the wrist) being proposed to estimate distal temperature [4,29,30]. However, limited literature data are available on how skin temperature recordings are affected when one or more sensors are missing.

Building on the equations proposed by Van Marken Lichtenbelt et al. [7], we have performed a simulation study which provides researchers and clinicians with: *i)* weights to modify the equations for temperature calculation in case one or more sensors are removed; *ii)* accuracy indicators of the goodness of the temperature estimates in case one or more sensors are removed; *iii)* guidelines on how to best position a reduced number of sensors, for example due to budget constraints or if subjects cannot tolerate the whole set of nine. All such data are formatted as two operative tables, one for proximal and one for distal skin temperature, supported by example reconstructions.

As far as proximal temperature *T*_*PROX*_ is concerned, the least accurate reconstruction occurred when the abdominal sensor was removed. This finding is somehow expected, since this sensor is the only one without a homologue (i.e. all the others have a right/left counterpart). Results show that, provided that weights are suitably re-tuned, a subset of three sensors positioned in any combination of three non-homologous regions (abdomen, right or left infra-clavicular and right or left mid-thigh) can still provide a reliable estimate of *T*_*PROX*_. With this subset, the MAE was around 0.1°C, thus comparable to the error intrinsic to each sensor.

As for the distal temperature *T*_*DIST*_, results show that accuracy of the estimates can be acceptable if at least one foot and one hand are included within a subset of three sensors, with the MAE in this situation being approximately 50% compared to that of either both feet or both hands included. Again, in this situation the MAE was around 0.1°C, therefore comparable to the error intrinsic to each sensor. The better accuracy observed when both hands were monitored (in a 3-sensor scenario, thus 2 hands and 1 foot as opposed to 2 feet and 1 hand) may be explained by the fact that hands are often separately exposed to the environment (for example when picking something from the fridge, or cooking) or moved separately from one another in daily life. In contrast, feet are more likely to be under similar environmental conditions, and generally mirror each other when moving or performing tasks. In the 2-sensor scenario, when two homologous sensors are chosen for reconstruction, performance suffers because of the compound effect of a low number of sensors, and limited information about the behaviour of sensors placed elsewhere in the body.

In conclusion, we provide results to support calculation and interpretation of proximal/distal temperature measurements obtained from any number of sensors (out of 9) on any combination of skin locations. These may be useful when temperature values are used to compare different groups of subjects, or to study changes in temperature over time or in relation to treatment.

## Supporting information

S1 File The pre-processing method used by Van Marken Lichtenbelt et al. [7] for wireless determination of skin temperature. (DOCX)
