# Supplementary material for: Expected accuracy of proximal and distal temperature estimated by wireless sensors, in relation to their number and position on the skin
Source: PLoS One. 2017 Jun 30;12(6):e0180315. doi: 10.1371/journal.pone.0180315 (PMC5493382; doi:10.1371/journal.pone.0180315)
Supplement: S1 File — (DOCX) [file pone.0180315.s001.docx]

**S1. Supporting information**

Van Marken Lichtenbelt et al. [7] originally proposed a pre-processing method aimed at compensating the propensity of the sensors to generate artifact. This was subsequently applied by Jones et al. [30], leaving out the final moving average step.

Formally, let x(t) denote a sample of the raw signal acquired by an iButtons^®^ temperature logger. Under the assumptions of uniform sampling and lack gaps in the data, the Rate of Change of x(t) is defined as the first difference signal ROC(t) := x(t+1) – x(t). The first and third quartiles of ROC(t), i.e. the values corresponding to the 25^th^ (Q1) and 75^th^ (Q3) percentile of ROC are then computed together with the interquartile range IQR = Q3 - Q1. At this point, only samples x(t*) for which ROC(t*+1) is between Q1- 1.5×IQR and Q3+1.5×IQR are retained whereas the rest are considered outliers. Clearly, as ROC(t) is one sample shorter than x(t), x(1) can never be filtered out this way. After this first step, Q1, Q3 and IQR are computed for the remaining values of x(t) and only the samples x(t*) between Q1- 1.5×IQR and Q3+1.5×IQR will be part of the final signal. In Van Marken Lichtenbelt et al. [7], at this point an 11-point rectangular smoothing is applied to the filtered signal x(t) to close the gaps. In the present study, this last step was omitted because it may have opened the way to uncontrolled error propagation which, in turn, could have negatively affected the final results.
